# Insight Between the Epigenetics and Transcription Responding of Cotton Hypocotyl Cellular Elongation Under Salt-Alkaline Stress

**DOI:** 10.3389/fpls.2021.772123

**Published:** 2021-11-11

**Authors:** Cun Rui, Yuexin Zhang, Yapeng Fan, Mingge Han, Maohua Dai, Qinqin Wang, Xiugui Chen, Xuke Lu, Delong Wang, Shuai Wang, Wenwei Gao, John Z. Yu, Wuwei Ye

**Affiliations:** ^1^State Key Laboratory of Cotton Biology/Institute of Cotton Research of Chinese Academy of Agricultural Sciences/Zhengzhou Research Base, School of Agricultural Sciences, Zhengzhou University/Key Laboratory for Cotton Genetic Improvement, MOA, Anyang, China; ^2^Crop Germplasm Research Unit, Southern Plains Agricultural Research Center, United States Department of Agriculture-Agricultural Research Service (USDA-ARS), College Station, TX, United States; ^3^Engineering Research Centre of Cotton, Ministry of Education/College of Agriculture, Xinjiang Agricultural University, Ürümqi, China

**Keywords:** *Gossypium barbadense*, cell elongation, DNA methylation, high pH alkaline, responding

## Abstract

*Gossypium barbadense* is a cultivated cotton not only known for producing superior fiber but also for its salt and alkaline resistance. Here, we used Whole Genome Bisulfite Sequencing (WGBS) technology to map the cytosine methylation of the whole genome of the *G. barbadense* hypocotyl at single base resolution. The methylation sequencing results showed that the mapping rates of the three samples were 75.32, 77.54, and 77.94%, respectively. In addition, the Bisulfite Sequence (BS) conversion rate was 99.78%. Approximately 71.03, 53.87, and 6.26% of the cytosine were methylated at CG, CHG, and CHH sequence contexts, respectively. A comprehensive analysis of DNA methylation and transcriptome data showed that the methylation level of the promoter region was a positive correlation in the CHH context. Saline-alkaline stress was related to the methylation changes of many genes, transcription factors (TFs) and transposable elements (TEs), respectively. We explored the regulatory mechanism of DNA methylation in response to salt and alkaline stress during cotton hypocotyl elongation. Our data shed light into the relationship of methylation regulation at the germination stage of *G. barbadense* hypocotyl cell elongation and salt-alkali treatment. The results of this research help understand the early growth regulation mechanism of *G. barbadense* in response to abiotic stress.

## Introduction

DNA methylation is an extensively studied epigenetic modification, which plays an important role in regulating gene expression and chromatin conformation. In general, high levels of DNA methylation can inhibit gene expression, and demethylation can trigger re-expression of genes. DNA methylation is involved in many activities of a living cell, including cell differentiation, tissue-specific gene expression, genome imprinting, X chromosome inactivation and other activities (Reik et al., 2001; Cedar and Bergman, 2012). There are three types of methylation in plants: CG, CHG, and CHH (where H = A, T or C). In the process of plant DNA replication, the methylation pattern of CG and CHG can be maintained by recognizing methylation marks, but the methylation pattern of CHH cannot be established through a similar recognition process (Law and Jacobsen, 2010). CHH methylation is guided by a small interfering RNA (siRNA) processed from the precursor RNA by Dicer-like 3. The methyltransferases *DRM1* and *DRM2* are the main reason for asymmetric methylation of CHH (Matzke and Mosher, 2014). More studies have shown that DNA methylation regulates the response mechanism of biotic stress (Dowen et al., 2012) and abiotic stress (Lu et al., 2017; Xu et al., 2018) by affecting the interaction between protein and DNA. DNA methylation plays an important role in plant stress response mechanism.

Various abiotic stresses, such as drought, high temperature, cold, salt, alkali stress, can affect the growth of plants, and the toxic effects of high pH can also cause serious damage to plants. First, high pH will cause changes in the state of minerals in the soil, which will further affect the physiological and ecological changes in the roots of the plant, and in severe cases will cause changes in the shape of the roots or even loss of function (Zhao et al., 2016). Studies have found that under acidic conditions, plant cells grow and extend quickly. When the intercellular pH increases to become alkaline, the cell wall’s loosening process is hindered, which affects cell extension. Experiments have confirmed that increasing the intracellular pH value will inhibit the growth of plant root hairs (Cosgrove, 2000). The types of soluble osmotic adjustment substances are tissue-specific, and there are differences among plant species. Many osmotic adjustment substances are accumulated under drought, high and low temperature. Research has found that 14-3-3 protein selectively binds to and inhibits PKS5 kinase activity by sensing calcium signals under saline-alkaline stress, thereby activating the activity of plasma membrane H^+^-ATPase and adapting plants to saline-alkali stress (Yang et al., 2019b). Researchers found that *SCaBP3/CBL7* lack of function plants have increased tolerance to alkaline stress, which is related to increased plasma membrane H^+^-ATPase activity. A central component of *Arabidopsis* alkaline tolerance is revealed as a Ca^2+^ sensor/kinase/plasma membrane H^+^-ATPase signal module that can adjust the stress response and fine-tune the plasma membrane proton flux (Yang et al., 2019a).

Numerous studies have shown that epigenetic factors play a very important role in the transcription of gene expression and post-transcriptional regulation (Mirouze and Paszkowski, 2011). While DNA methylation and DNA sequence co-evolve, the speed of DNA methylation evolution is much greater than that of DNA sequence. Therefore, in comparison with DNA sequence, DNA methylation can respond faster to environmental changes (Song et al., 2017). In *Arabidopsis*, many genes that respond to phosphate starvation are demethylated in its upstream region (Yong-Villalobos et al., 2015). A large number of studies have shown that various adversities, such as heat stress, salt stress, drought, will affect DNA methylation levels, and many genes regulated by DNA methylation are often related to environmental stress responses, indicating chemical dynamics of that DNA methylation participates in the regulation of gene expression under environmental stress (Ma et al., 2018; Xu et al., 2018). In rice (*Oryza sativa*), methylation variation in the promoter region inhibits gene expression, but methylation in the main gene region is associated with gene expression (Feng et al., 2016). External environmental stress can cause epigenetic changes at the TE site in the plant genome, contributing to the regulation of the transcription of TEs in response to stress (Sahu et al., 2013). Overall, epigenetics contributes to regulatory in plant stress response.

As one of the important sources of fiber and edible oil, cotton has great economic value. Many studies have shown that DNA methylation is closely related to the growth and development of cotton. The regulation mechanism of DNA methylation at three stages of cotton growth (seedling stage, presquaring stage, and squaring stage) were analyzed, finally the key circadian rhythm regulator, homology of cryptochrome (CRY), LATE ELONGATED HYPOCOTYL (LHY), CONSTANS (CO) were discovered, gene expression is related to changes in DNA methylation at the three stages of development (Sun et al., 2018). Studies have found that high temperature stress during the development of cotton anthers can cause epigenome changes, such as DNA methylation and histone modification (Min et al., 2014). DNA demethylation can promote the development of anthers, while the increase in methylation level only partially inhibits the development of anthers under high temperature stress (Zhang et al., 2020). The study on the process of somatic embryogenesis of cotton and the leaves of regenerated progeny plants [non-embryogenic callus (NEC), EC, and somatic embryos (SE)], showing that induced hypomethylation of successful regeneration acclimation (SRA) may promote higher plant regeneration capacity and optimize maternal genetic varieties (Li et al., 2019). Because current reports on cotton mainly focus on salt-alkaline stress, insufficient understanding of the relationship remains between salt-tolerant mechanisms and ion groups. In the steady-state changes of cotton ions, Na^+^ is the key ion that affects the salt (alkaline) tolerance mechanism of cotton. Beginning with Na^+^ to study its molecular mechanism will directly help to further understand the changes in ion steady-state under high pH environment. With few reports available on *G. barbadense* alkaline tolerance mechanism, they mainly focus on salt-alkali stress, and little research is reported on *G. barbadense* alkaline tolerance mechanism. Therefore, it is necessary to investigate *G. barbadense* response to methylation for regulating cell elongation under alkaline stress.

In this study, we constructed a single-base resolution and genome-wide map of *G. barbadense* cytosine methylation through Whole Genome Bisulfite Sequencing (WGBS) technology, which included the methylation level of the *G. barbadense* genome in response to salt and alkaline stress. Through integrated analysis with RNA-seq sequencing results, this study was to understand: (1) the genomic landscape of the *G. barbadense* hypocotyl methylome, (2) methylome changes in the *G. barbadense* associated with salt and alkali stress, (3) the relationship between methylome changes and salt and alkali stress-associated gene expression changes, and (4) the epigenetic mechanism of *G. barbadense* hypocotyl elongation under alkaline stress.

## Materials and Methods

### Plant Materials and Sample Collection

The seeds of *G. barbadense* alkali-tolerant material Jiza 67 were was used for this study. Full-grained seeds were selected and disinfected 75% alcohol for 30 s, and the treatment were repeated for 3 times (the treatment process was properly shaken to ensure that the seeds are in full contact with the alcohol). The treated seeds were rinsed with distilled water for 5 times. They were wrapped in filter paper for germination in the dark at 28°C for 2 days. Then, the seedlings with consistent germination and growth were selected and treated with 50 mM Na_2_CO_3_ and 100 mM NaCl stress, respectively. The control group was wrapped in filter paper soaked in distilled water. After 12 h, the hypocotyls of the young shoots were quickly frozen in liquid nitrogen for 30 min and stored in an ultra-low −80°C freezer. Based on different treatments, the control sample was designated as CK, the Na_2_CO_3_ processed sample designated as Alk, and the NaCl processed sample designated as Sal. Three biological replicates were applied for each treatment.

### DNA and RNA Extraction and Library Construction

Total genomic DNA of hypocotyls was extracted according to the improved CTAB extraction protocol. The DNA samples fragmented by sonication were subjected to bisulfite conversion. The Accel-NGS Methyl-Seq DNA Library Kit (Swift, MI, United States) was used for attaching adapters to single-stranded DNA fragments. Briefly, the adaptase step was a highly efficient, and proprietary reaction that simultaneously performs end repair, tailing of 3′ ends, and ligation of the first truncated adapter complement to 3′ ends. The extension step was taken to incorporate truncated adapter 1 by a primer extension reaction. The ligation step was taken to add the second truncated adapter to the bottom strand only. The indexing PCR step was taken to increase yield and incorporate full length adapters. Bead-based SPRI clean-ups were used to remove both oligonucleotides and small fragments, as well as to change enzymatic buffer composition. Finally, we performed the pair-end 2 × 150 bp sequencing on an Illumina Hiseq 4000 platform (LC Sciences, San Diego, CA, United States) with sequencing depth of 30×.

Transcriptome sequencing was performed on three biological replicates that were consistent with the methylation sequencing. The total RNA was extracted using Trizol reagent (Invitrogen, Carlsbad, CA, United States) following the manufacturer’s instructions. The quantity and purity of the RNA were analyzed with Bioanalyzer 2100 and RNA 6000 Nano Lab Chip Kit (Agilent, CA, United States) with RIN number > 7.0. Then approximately 10 μg of total RNA was purified using poly-T oligo-attached magnetic beads and the purified RNA was cleaved into smaller fragments with fragmentation buffer. PCR amplification was performed to obtain the final sequencing library. The average insert size for the paired-end libraries was 300 bp (±50 bp). Paired-end sequencing was performed on an Illumina Novaseq^TM^ 6000 (LC Sciences, San Diego, CA, United States) following the manufacturer’s protocols.

### RNA Sequencing Analysis

The website^1^ was used to estimate the expression level of all transcripts and perform it for mRNAs by calculating fragments per kilobase of exon model per million mapped fragments (FPKM). Gene ontology (GO) enrichment and kyoto encyclopedia of genes and genomes (KEGG) enrichment were analyzed to the differentially expressed genes (DEGs). The GO seq R package (Young et al., 2010) was used for GO enrichment analysis and KOBAS software was used to test the statistical enrichment of the differentially methylated genes (DMGs) in KEGG pathways (Kanehisa et al., 2008). GO terms or KEGG pathways with a corrected *P*-value < 0.05 were considered significantly enriched.

### Methylation Data Analysis

Cutadapt (Mirouze and Paszkowski, 2011) and in-house perl scripts were used to remove adapter sequences, low quality bases and undetermined bases. Then sequence quality was verified using Fast QC^2^. Reads that passed quality control were mapped to reference genome using Bismark (Krueger and Andrews, 2011). After the alignment, reads were further deduplicated using samtool (Li et al., 2009). For each cytosine site (or guanine corresponding to a cytosine on the opposite strand) in the reference genome sequence, the DNA methylation level was determined by the ratio of the number of reads supporting C (methylated) to that of total reads (methylated and unmethylated) using the in-house perl scripts and Meth Pipe (Song et al., 2013). Differentially methylated regions (DMRs) were calculated by R package-Methyl Kit (Akalin et al., 2012) with default parameters (1,000 bp slide windows, 500 bp overlap, adjust *P*-value < 0.05). Concerning the relationship between DNA methylation and gene transcription regulation, we set genes with gene expression value fpkm < 1 as none (no expression) level; rank fpkm values of genes with fpkm > = 1 from small to large, calculate the fpkm values fpkm_25% and fpkm_75% at the upper and lower quartiles; genes with 1 ≤ fpkm < fpkm_25% listed as low grade; genes with fpkm_25% ≤ fpkm < fpkm_75% as medium grade; put fpkm ≥ fpkm_75% of genes were classified as high grade.

### Making Paraffin Sections

The hypocotyl sample was placed in FAA fixative for 2 days. The alcohol dehydration method was used for dehydration embedding, and section processing. The hypocotyl sample was dewaxed, dyed with safranin staining solution, dehydrated with ethanol, and sealed with glue. Finally microscopic examination and pictures were taken with. FcSnap software was used to measure cell length. A total of 50 cell lengths were measured with three replicates.

### Real-Time Fluorescence Quantification of Differentially Expressed Genes

The cDNA was prepared According to TransScript II All-in-One First-Strand cDNA Synthe-sis SuperMix for qPCR (One-Step gDNA Removal) instructions. The equipment used for qRT-PCR was Applied Biosystems@ 7500 Fast, and the fluorescence quantification kit was TransStart Top Green qPCR SuperMix. The ΔΔ*C*t method (Zhang et al., 2006) was used to calculate the processing results.

## Results

### Elongation of *G. barbadense* Hypocotyl Under High pH Alkaline Stress

**FIGURE 1:**
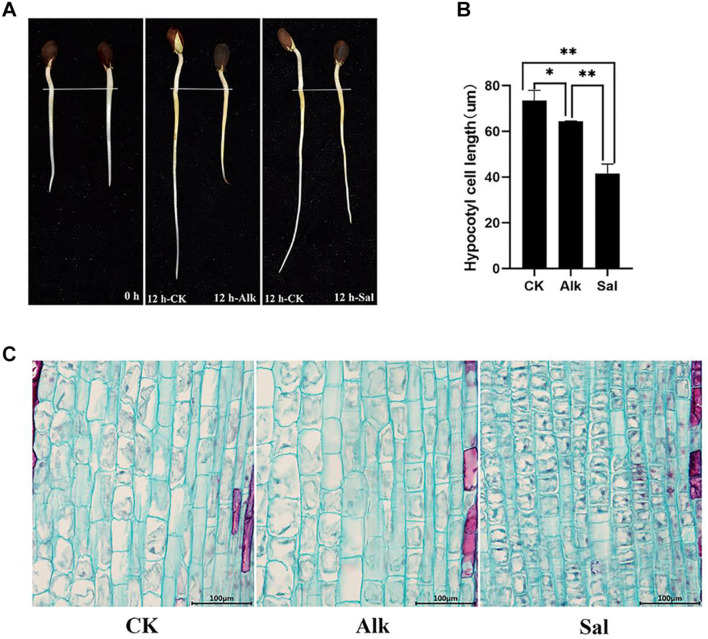
The elongation characteristics of *Gossypium barbadense* hypocotyl cells under CK, Alk and Sal stresses. **(A)** The growth of *G. barbadense* hypocotyl under alkaline stress, from left to right are 0 and 12 h. **(B)** Statistics of the cell length of the three samples. Epidermal cell length was measured with the FcSnaP software and the lengths were averaged from at least 50 cells. The statistical significance of the measurements using one-way analysis of variance (ANOVA) was determined with test. The asterisk indicates the significant difference between the two stresses and the control group (*P < 0.05, **P < 0.01). **(C)** Hypocotyl cell length under salt and alkaline stress, Scale bars, 100 μm.

The tender shoots of cotton seedlings were treated with high pH alkaline (Na_2_CO_3_) and sodium salt (NaCl) for 12 h (Figure 1A). It was speculated that sodium salt and high pH alkaline stress likely inhibited *G. barbadense* hypocotyl elongation. To further understand the effect of the two stresses on the elongation of hypocotyl cells, we observed the cell morphology (Figure 1C) by cell slicing. By measuring the cell length, we found that the length of the hypocotyl cells after stress was significantly shorter than that of the control. At the same time, we learned from the data that the two treatments inhibited the cell elongation of the hypocotyl, but the cell length between the two treatments also changed significantly (Figure 1B). Such variation may be due to different responses of high pH alkaline (Na_2_CO_3_) and sodium salt (NaCl) to cell elongation.

### Methylation Landscapes in *G. barbadense*

**FIGURE 2:**
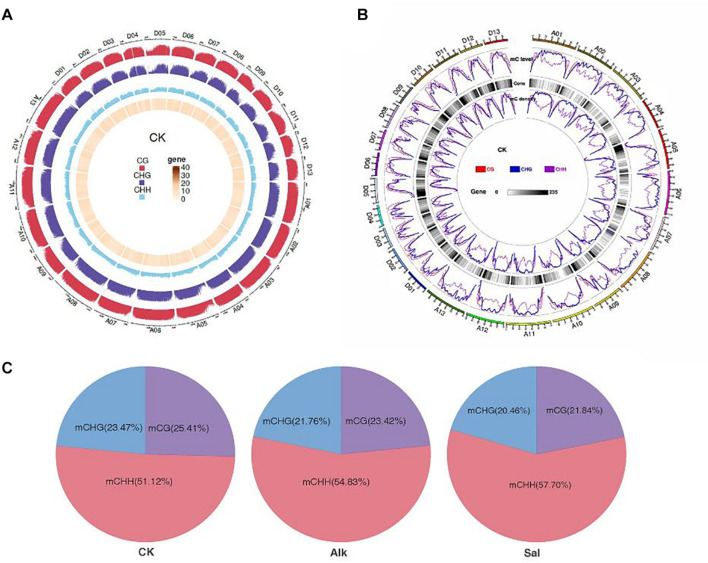
Epigenome of *G. barbadense.* **(A)** Circle graph of the methylation distribution within chromosomes. The outermost circle is a scale based on the length of the corresponding chromosome. The following three circles (from outside to inside) represent the methylation background display of CG, CHG, and CHH, respectively, in the corresponding chromosome interval of the PF group in red, purple, and blue, respectively. The denser color line indicates the higher level of methylation background. The innermost circle indicates the number of genes in the corresponding interval, and the darker color indicates the greater number of genes in the region. **(B)** Circos plot showing the level and density of 5-mCs in the three sequence contexts and the gene density for all 26 chromosomes of the *G. barbadense* genome. For the line graph of the level and density of 5-mCs, the CG, CHG, and CHH sequence types are displayed as red, blue, and purple lines, respectively. **(C)** Relative proportions of mCs in CG, CHG, and CHH contexts across the genome of *G. barbadense* hypocotyl.

To study the dynamic changes of hypocotyl DNA methylation during *G. barbadense* germination were observed under salt-alkaline stress, WGBS technology was used to develop a genome-wide single-base resolution map of *G. barbadense* hypocotyl cytosine methylation (Figure 2A), and the changes of *G. barbadense* methylation patterns were analyzed under the two stresses. The single-base resolution diagrams of Alk and Sal samples are shown in Supplementary Figures 1A,B. The mapping rate of CK, Alk and Sal was 75.32, 77.54, and 77.94%, respectively (Table 1), based on the sequencing data. 52,498,262, 386,850,350, and 462,494,704 reads were obtained from control check (CK), alkaline (Alk) and salt (Sal) stresses, respectively. The CK displayed approximately 19.74, 71.03, 53.87, and 6.26% methylation in the C, CG, CHG, and CHH contexts. Alk was about 22.67, 74.91, 58.78, and 8.17% methylation and Sal was about 23.1, 75.02, 59.12, and 8.72%, respectively. The data showed that under the two treatments, the methylation levels of the three sequences of CG, CHG, and CHH all increased, and that of the CHH sequence increased most. Based on the relative mC proportions of CG, CHG, and CHH in the *G. barbadense* hypocotyl (Figure 2C and Table 2), the CHH sequence accounts for a largest proportion. To understand the specific distribution of CG, CHG, and CHH on each chromosome in more detail, we drew a circle map of the methylation sequencing data showing the zone distribution within the chromosome. The level and density of 5-mCs among the three sequence contexts and the gene density for all 26 chromosomes of the *G. barbadense* genome are shown in Figure 2B. Similarly, the sequence density diagrams of Alk and Sal samples are shown in Supplementary Figures 1C,D.

**TABLE 1:** Statistical results of methylated cytosines in different contexts.

Samples	Total reads	Mapped reads	Mapping rate (%)	mC percent (%)	mCG percent (%)	mCHG percent (%)	mCHH percent (%)
CK	467 992 658	352 498 262	75.32	19.74	71.03	53.87	6.26
Alk	498 926 470	386 850 350	77.54	22.67	74.91	58.78	8.17
Sal	593 381 376	462 494 704	77.94	23.1	75.02	59.12	8.72

*Total reads are the clean sequence reads after the data filtration. Mapping rate is the reads with unique positions on the reference genome after alignments. Percent (%) of mC, mCG, mCHG, and mCHH is calculated with the methylated cytosines in each context of all methylated cytosines in the whole genome.*

**TABLE 2:** Summary of WGBS reads for two different stress conditions.

Samples	Duplication rate (%)	BS conversion rate (%)	mC percent (%)	mCG percent (%)	mCHG percent (%)	mCHH percent (%)
CK	10.83	99.78	263452823 (100)	66948111 (25.41)	61821662 (23.47)	134683050 (51.12)
Alk	12.87	99.78	285743214 (100)	66909154 (23.42)	62171921 (21.76)	156662139 (54.83)
Sal	14.26	99.78	309525090 (100)	67605546 (21.84)	63321411 (20.46)	178598133 (57.70)

*Duplication rate is the percentage of repetitive sequences among all clean sequencing reads. Each percent (%) of mCG, mCHG, and mCHH is the percentages of methylated mCG, mCHG, and mCHH in all corresponding cytosine contexts (C, CG, CHG, and CHH and H represents A, C, or T) in the genome, respectively.*

### Preference of Cytosine Sequence Under Salt and Alkaline Stress

**FIGURE 3:**
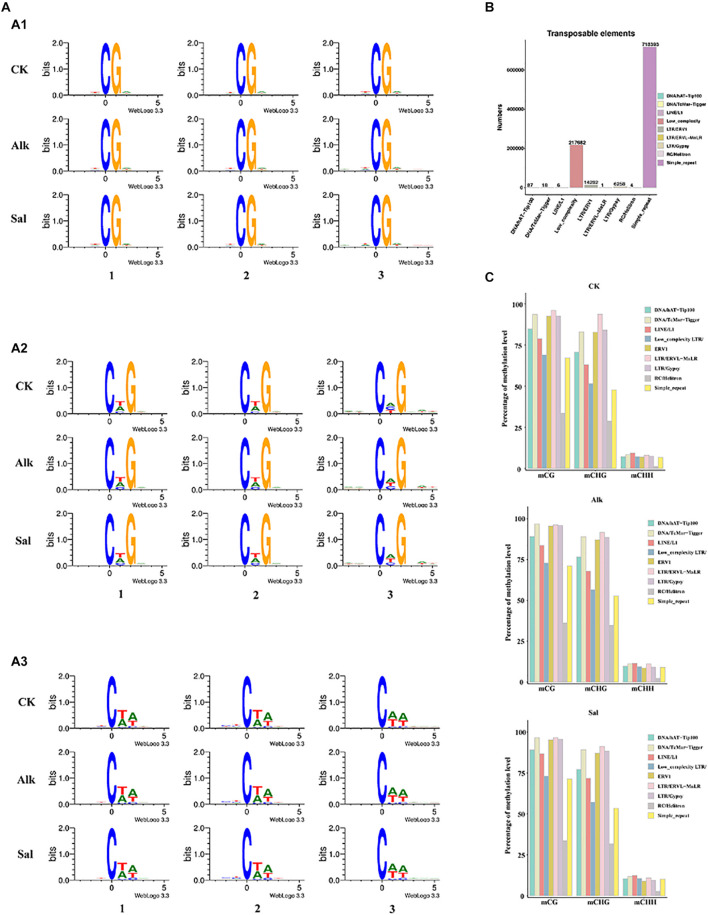
Sequence preference of cytosine DNA methylation and methylation patterns of (TEs) in the *G. barbadense* genome. **(A)** Sequence preference of cytosine DNA methylation and differentially methylated cytosines in gene elements. (x) Weblogo analysis of bases around 5-mCs in CG (x), CHG (x), and CHH (x) sequence contexts. The *x-*axis represents the position of 9-bp sequence information near the 5-mCs; the *y*-axis represents the conservation of bases at the site, A1, A2 and A3 represent CG, CHG and CHH context, respectively. 1 represent all mC sites, 2 represent high methylation sites (defined >75% methylation level), 3 represent low methylation sites. In non-CG context, high methylation level sites were defined methylation level >25% and others were low methylation level sites. **(B)** Numbers of different types of TEs in *G. barbadense* genome. **(C)** Percentage of methylation levels of different types of TEs in three samples.

Occurrence of cytosine methylation was previously reported as highly associated with its DNA sequences (Bock et al., 2006; Lister et al., 2008). To determine the sequence preference around all mCs in *G. barbadense* hypocotyls, we used Logo Plots to analyze the sequence information near the methylation sites in different sequence backgrounds (Figure 3A). We found that in the symmetrical CG sequence context, mC sites often appear on the TCGA sequence, and there is no tendency to change with the level of methylation. In the CHG sequence context, mC sites with high methylation levels mostly appear in CTG sequences, while at low methylation levels, mC sites often appear in CAG sequences. Similarly, we found that the high-level and low-level mC sites in the CHH sequence context occurred at the CTA and CAA sequences, respectively. In view of the above results, in the context of CHG and CHH sequences, high and low levels of mC mostly seem to occur at CT- and CA- sequences, respectively. As the methylation level changes, the sequence of mC sites would also change (Chen et al., 2011).

### Statistics of Methylation Distribution of Different Gene Segments

**FIGURE 4:**
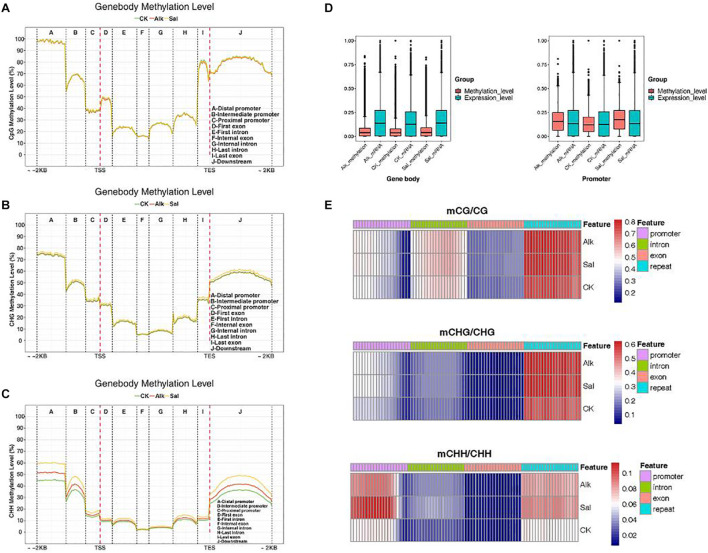
DNA methylation patterns in different genomic regions. **(A–C)** Statistics of methylation distribution of different gene segments. The *X*-axis represents different regions of the genome. **(D)** Box plots of methylation levels and gene expression levels in gene bodies and promoter regions [FPKM value was estimated by log10 (FPKM + 1)/max log10 (FPKM + 1)]. **(E)** Analysis of the heat map of the methylation level of gene regions.

We made statistics on the average of methylation levels in the whole genome of each sample and developed a violin chart (Supplementary Figure 2). To clarify the methylation changes of functional elements on the genome, for the three samples, we selected a series of methylation patterns of functional elements for further analysis. We counted the average methylation levels of C sites in various genomic functional regions [2 kb upstream of the transcription start site (TSS) to 2 kb downstream of the transcription end site (TES) in each context]. We divided it into promoter, exon, intron, and downstream. As shown in Figures 4A–C, the methylation level in the promoter region was higher, that level in the exon region began to decline, it gradually increased in the last intron region, and finally rose to a high level in the last exon and downstream regions. In each gene element, the frequency of various backgrounds including CG methylation, CHG methylation and CHH methylation was different. Then, the methylation levels of promoters, exons, and introns were compared in different contexts before and after salt-alkali stress, and only slight changes were found. The distribution data of methylation levels in functional regions were used to draw heat maps for showing the methylation levels of the three samples (Figure 4E).

In the three different sequence contexts, the methylation levels of functional elements on the genome of the samples of the treatment group were lower than those of the control group, compared with the control, the methylation level changed after the stress. Plant regulated gene expression by changing the methylation level during adversity, thereby responding to the adversity response. In the CG and CHG sequence contexts, the three samples had no significant difference in methylation levels, but in the CHH sequence context, the methylation levels of each sample in the promoter and downstream regions were quite different. The levels of methylation were the lowest in the control group, and higher in salt stress than alkaline stress. This may be due to the difference in salt and alkaline stress effects. Studies have shown that DNA methylation can cause changes in chromatin structure, DNA conformation, DNA stability and the way that DNA interacts with proteins, so as of controlling gene expression (Harris et al., 2018). We found that the methylation level of the promoter initiation region was significantly higher than that of exons and introns. As shown in Figure 4D, we analyzed the methylation level and transcription level of the gene body and the promoter region, and we found that the methylation level of the promoter region was higher than the methylation level of the gene body, and the transcription level of the promoter region was related to the methylation level. These data indicated that DNA methylation was at least partly responsible for the transcriptional changes in the promoter regions of these genes. A portion of DEGs was not directly targeted by DNA methylation, but they were differentially expressed due to methylation-dependent changes in the transcription network.

### Correlation Between DNA Methylation and Gene Expression Levels in *G. barbadense* Hypocotyls

**FIGURE 5:**
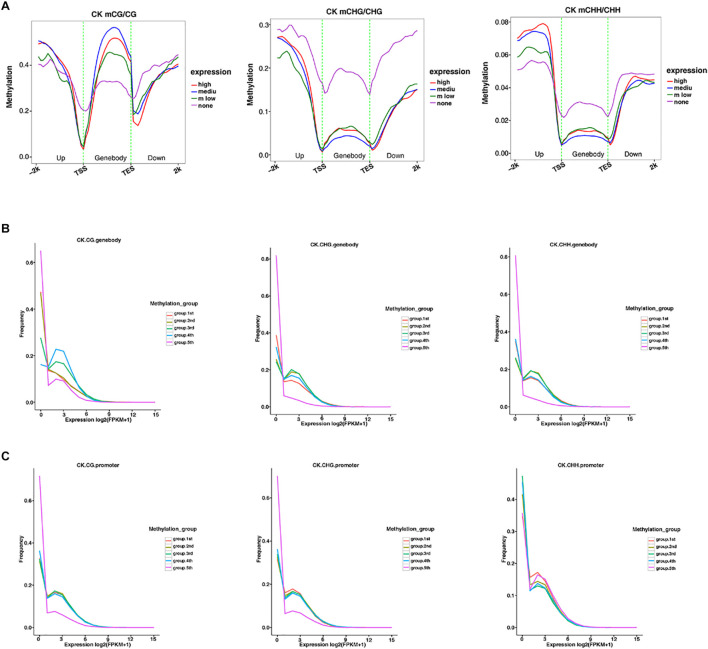
The relationship between methylation among different expression profiles and the overall transcriptome. **(A)** The distribution of methylation levels of genes under different expression levels in the gene body and its upstream and downstream 2 Kb regions under different sequence contexts (the abscissa represents different regions, and the ordinate represents the methylation level. Different colors represent different expression levels). **(B,C)** The expression levels of genes under different methylation levels in the gene body and promoter regions under different sequence context. (The abscissa represents the expression level, and the ordinate represents the frequency of the gene at the corresponding expression level. Different colors represent different methylation grade).

The methylation level distribution of gene body and its upstream and downstream regions were mapped in different sequence contexts (Figure 5A and Supplementary Figures 3A, 4A). The same mapping was conducted to classify the methylation level, and the three samples were plotted to reveal the relationship between the methylation level and transcription expression (Figures 5B,C and Supplementary Figures 3B,C, 4B,C). Under the CG context, the methylation level of the gene body was higher than that of the CHG and CHH sequence contexts, respectively. Similarly, the transcription starting site and the termination site showed low methylation levels in the three contexts. Moreover, in the CHG and CHH sequence contexts, the medium-level genes exhibited low methylation levels, which was the opposite of the trend in the CG context. Analysis of the relationship between promoter and gene frequency of genes with different methylation levels (Figures 5B,C), showed that the frequency of all genes decreased with the increase of expression level. Interestingly, in the fifth group, the expression level of high methylation level gene promoters in the CHH sequence context seemed to be positively correlated with the methylation level.

### Transposon and Methylation Level in *G. barbadense*

Transposable elements can affect the size of the genome, and they can generate mutations through insertion and excision, which affect gene expression. To describe the methylation characteristics of TE, we first identified members of different TE types in the *G. barbadense* hypocotyl genome (Figure 3B). Among them, the three types of simple-repeat, low-complexity and LTR/ERV1 were the most abundant. Then we analyzed the methylation levels of different types of TEs. In the two sequence contexts of CG and CHG, TEs showed hyper methylation levels, while in the CHH sequence contexts, different types of TE methylation levels varied little, and all showed hypo methylation levels. For all different types of TEs, RC/Helitron had the lowest methylation level, DNA/TcMar-Tigger, LTR/ERV1, LTR/ERVL-MaLR, LTR/Gypsy all showed hyper methylation levels (Figure 3C). Low-complexity TE is an uncommon type. In our data, its methylation level had no obvious characteristics. While the methylation level of TE was reported as likely related to the length of TE (Xu et al., 2018), the relationship between TE length and methylation level in the hypocotyl of *G. barbadense* at the germination stage needs to be further investigated.

### Enrichment Analysis of Differentially Methylated Genes

To determine the functional difference between methylated and unmethylated genes in this study, we used GO analysis to classify methylated and unmethylated genes (Supplementary Figures 5A–C). We classified genes into three aspects: biological process, cellular component, and molecular function. For biological process, methylated genes were mainly enriched in mRNA splicing, via spliceosome and protein polyubiquitination, the unmethylated genes were mainly enriched in the flavonoid biosynthetic process and the proteasome mediated ubiquitin dependent protein catabolic process. For cellular component and molecular function, whether genes were methylated or not, they were enriched in nucleus, cytoplasm, DNA binding and protein binding. To further understand the enrichment of these two pathways, we established a KEGG pathway enrichment analysis. As shown in Supplementary Figures 5D–F, most of the unmethylated genes were enriched in RNA transport, endocytosis and oxidative phosphorylation pathways, while the methylated genes were concentrated in plant hormone signal transduction, MAPK signaling pathway, and plant-pathogen interaction pathways. This was essentially consistent with the relationship between methylation level and gene expression level.

### Dynamic Model of Differential DNA-Methylation Level in Response to Salt and Alkaline Stress

**FIGURE 6:**
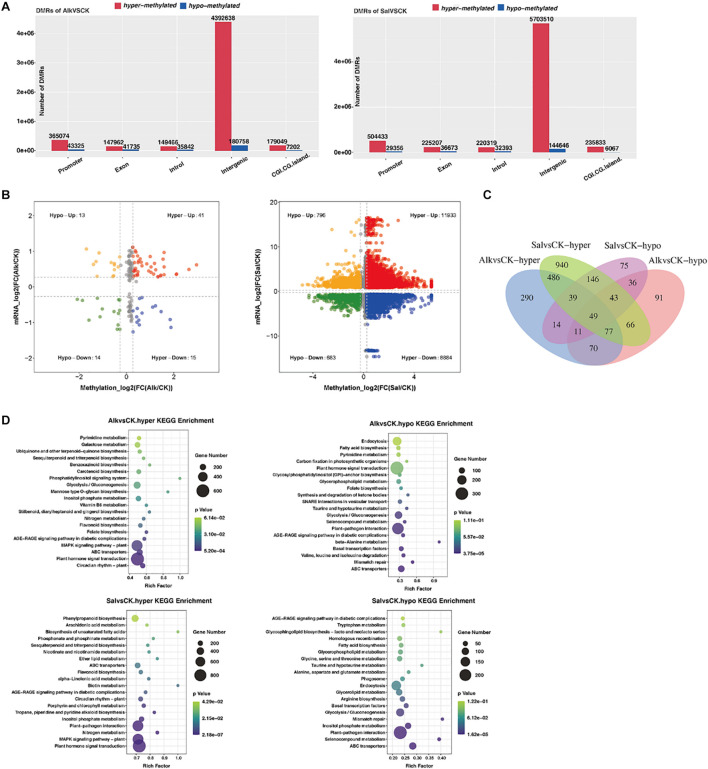
Analysis of methylation level and enrichment between samples. **(A)** Histogram of gene structure statistics of methylation genes. The *x*-axis represents the functional regions of different genes, the *y*-axis represents the number of DMRs, red represents up-regulated DMR, and blue represents down-regulated DMR. **(B)** Four-quadrant diagram of DNA methylation and mRNA. The two groups are drawn on the basis that all points are less than 0.05, or, the *P*-values of all points are less than 0.05. One point represents a DMR area, the log2 of -inf is 0, and the maximum value of inf is added to 0.00001 and then taken log2 value. **(C)** Venn diagram of hyper/hypo methylated genes among *G. barbadense* under salt and alkaline stress. **(D)** KEGG pathway enrichment of hypermethylated and hypomethylated genes in *G. barbadense* under salt and alkaline stress. The size of the circle represents gene numbers, and the color represents the *P*-value. Alk vs. CK, alkaline stress versus control; Sal vs. CK, salt stress versus control.

To understand the dynamic effects of salt and alkaline stress on methylation, we analyzed the DMRs of the two treatment groups and the control group. In the promoter region, 365074 and 504433 hypermethylated DMRs were identified in alkaline stress versus control (Alk vs. CK) and salt stress versus control (Sal vs. CK), respectively, while 43,325 and 29,356 hypomethylated DMRs were identified (Figure 6A and Supplementary Table 1). In the exon region, we identified 147,962 and 225,207 hypermethylated DMRs in Alk vs. CK and Sal vs. CK, respectively, and 41 735 and 36 673 hypomethylated DMRs were simultaneously identified. Also, the number of hypermethylated DMRs and hypomethylated DMRs was different in introns, intergenic and other regions under saline-alkaline stress. This result suggests that differences exist between the two abiotic stresses.

In comparison of the difference between groups, the methylation level of DMRs in each group was classified according to high and low levels, and statistics analysis was conducted. The Venn diagram (Figure 6C) shows that Alk vs. CK and Sal vs. CK shared 651 hypermethylated DMRs and 139 hypomethylated DMRs. To further understand the biological functions of the gene response after stress, we performed KEGG (Figure 6D) and GO (Supplementary Figures 6A,B) enrichment analysis on hypermethylated and hypomethylated genes. In Alk vs. CK and Sal vs. CK pairs, hypermethylated genes were mainly enriched in plant hormone signal transduction, while for hypomethylated genes, the former was mainly enriched in plant hormone signal transduction, and the latter was reflected in plant-pathogen interaction. Through the biological function of GO enrichment, we concluded that the hypermethylation genes in Alk vs. CK were mainly distributed in the pathways of DNA binding, protein binding, and protein serine/threonine kinase activity. In Sal vs. CK, the hypermethylation genes mainly distribute DNA-binding transcription factor (TF) activity and were enriched in metabolic pathways. The hypomethylation of the two treatment groups was enriched in the protein serine/threonine kinase activity pathway, and it was interesting that the hypomethylation genes in Sal vs. CK were also enriched in the ATP binding, Golgi membrane, and microtubule motor activity pathways.

The hypermethylation genes enriched in the plant hormone signal transduction pathway in Alk vs. CK and Sal vs. CK were mainly allocated to tryptophan metabolism, zeatin biosynthesis, brassinosteroid biosynthesis, and secondary biosynthetic pathways of terpenoids, metabolites. A large number of DMGs were involved in the plant hormone signaling pathway. Genes related to the hormone signaling pathway, including IAA, cytokinin, gibberellin (GA), abscisic acid (ABA), jasmonic (JA), and salicylic acid (SA) synthesis related genes, were also methylated. The expression and methylation level of Xyloglucan Endotransglucosylase/hydrolase (XTH) gene, which was closely related to cell elongation, changed significantly. A quadrant plot of the relationship between methylation level and gene expression (Figure 6B) indicated that DNA methylation was responsible for at least some of the transcriptional changes of genes. Some DEGs were not directly targeted by DNA methylation, but they were differentially expressed due to methylation-dependent changes in the transcription network. For abiotic stress, the methylation level of genes related to phenylalanine metabolism also changed. Based on the relationship between methylation and gene expression levels, DNA methylation levels appeared to affect the transcriptional expression of some genes.

### Widespread Dynamic DNA Methylation in Response to Salt and Alkaline Stress

**FIGURE 7:**
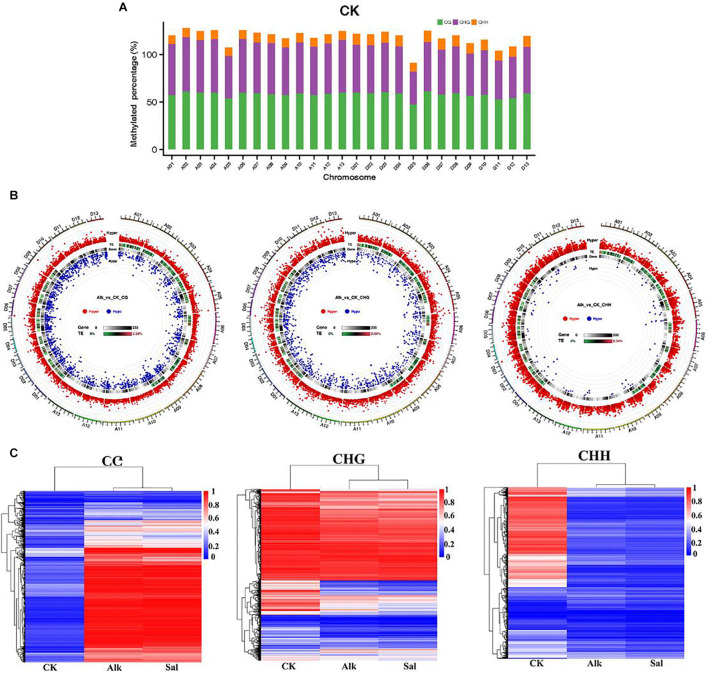
Dynamic DNA methylation in response to salt and alkaline stress. **(A)** Dynamic changes of DNA methylation under stress. Different colors represent methylated C sites under different contexts, and the length of each column represents the percentage of the sequence context methylation sites in the sequence context of the chromosome. **(B)** The overall circos diagram of DMR in three sequence contexts (CG, CHG, and CHH). Graphic display instructions, from the outside to the inside, with the scale indicated by the color: (1) hyper DMR statistical value log5 (| areaStat|); the higher the outer dot, the larger the position difference is, and it is indicated by the red circle, (2) TE original proportion heat map, (3) gene density heat map, (4) hypo DMR statistical value log5 (| areaStat|); the higher the inward dot, the more significant the position difference. **(C)** Heat maps of methylation levels within CG, CHG, and CHH DMRs, respectively.

To study the potential effects of salt and alkaline stress on methylation, we calculated the methylation level of each chromosome in the *G. barbadense* genome. The ratio of methylated C sites to the total C sites on each chromosome under different sequence contexts was shown in Figure 7A and Supplementary Figures 7A,B. The methylation level of CG, CHG, and CHH in the three sequence contexts of the 5 chromosome was lower than other chromosomes in the *G. barbadense* genome. To further understand the difference at methylation levels on each chromosome, through the distribution and significance of DMR on the genome, we analyzed the length distribution of the DMRs and those between Alk vs. CK and Sal vs. CK on each chromosome methylation level. Under the stress of two types of Na^+^, the number of hypermethylated DMRs in the three sequence contexts was greater than that of hypomethylated regions. Whether it was hypermethylated or hypomethylated regions, DMRs were mainly concentrated in CG and CHG sequence contexts (Figure 7B and Supplementary Figures 7A,C). In the CHH sequence context, the number of hypomethylated DMRs was the least. We performed correlation analysis on the DMRs of the three sequence contexts based on the methylation level to draw a heat map. The correlation heat map shows the changes in the methylome of *G. barbadense* hypocotyls under two stresses (Figure 7C). The heat map suggested that the methylation level of DMRs was highly correlated under two treatments in the CG and CHG sequence contexts.

### Regulation of DNA Methylation in Plant Hormone Signal Transduction Under Na^+^ Stress

**FIGURE 8:**
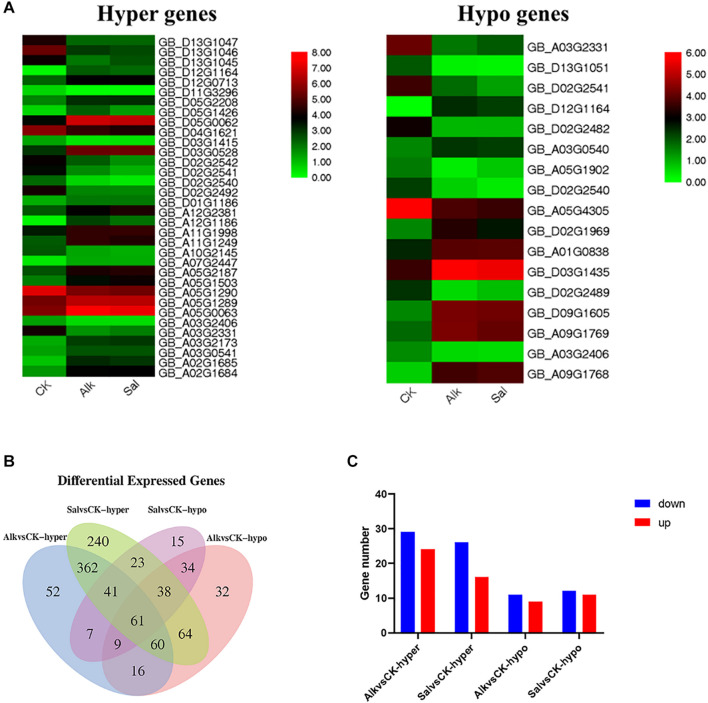
Analysis of the methylation and transcription levels of plant hormones in response to Na^+^ stress. **(A)** Heap maps of DEGs. **(B)** Venn diagram of DMR genes in plant hormone signal transduction under abiotic stress. **(C)** Gene numbers of DMRs in plant hormone signal transduction pathways.

Based on the enrichment results of GO and KEGG pathways, we identified hormone-related DEGs in both Na^+^ stresses (Figure 8B). Among them, there were 77 DEGs under high pH Na^+^ stress, and 100 DEGs were found in the other Na^+^ stress. At the same time, we screened the common differential genes of Alk vs. CK and Sal vs. CK based on the differential methylation level, and drew the heat map (Figure 8A). The results show that the genes that responded to two stresses at the same time had the same up-regulation relationship regardless of the methylation level. We suggest that the regulation of methylation on gene transcription may be the same in general. These co-expressed genes were mainly enriched in the biosynthesis of auxin, zeatin, gibberellin, brassinolide, diterpene esters, and carboxylesterase. Differential genes related to plant hormones (brassinolide, gibberellin, auxin, and zeatin) were selected to verify their expression levels. The results showed that the expression level of the selected gene was basically the same as that of RNA-seq (Supplementary Figure 8).

**FIGURE 9:**
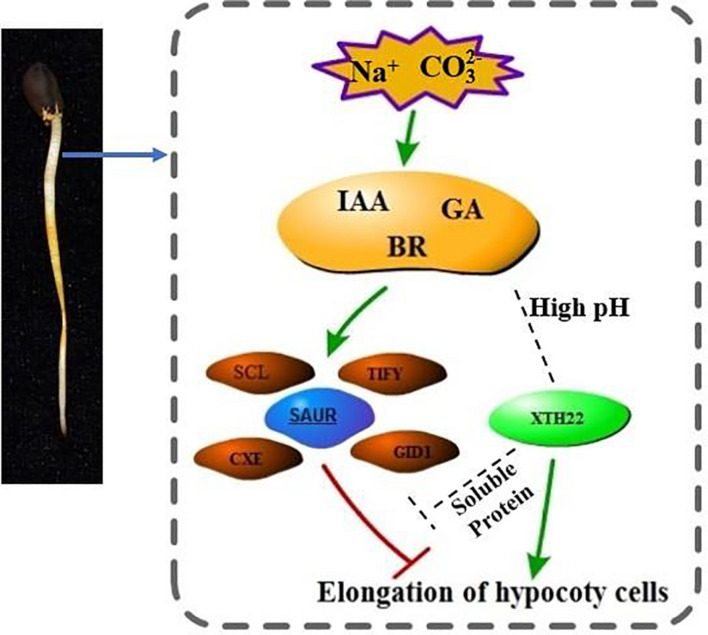
The regulation model of hormone-related genes involved in DNA methylation under Na^+^ stress. Under Na^+^ stress, DNA methylation mediates the transcriptional changes of related genes mainly related to IAA, GA, BR, including the up-regulation of SCL, TIFY transcription factors and stress response proteins such as CEX and CID1, and the expression of SAUR protein. The gene encoding XTH22 protein is substantially up-regulated under high pH environment. XTH22 protein interacts with a soluble protein, and it promotes cell elongation under the concerted action of hormones, thereby alleviating the inhibition of Na^+^ stress. The mechanism of interaction between XTH22 protein and hormones remains unclear, thus we use the gray dotted line to mark the status.

For the two stress-specific genes, we investigated the relationship between up and down regulations (Figure 8C). More hypermethylated genes were identified than hypomethylated differential genes in the specifically expressed genes. Interestingly, we found that in the hypomethylation genes specifically expressed in the Alk vs. CK group, the expression of the xyloglucan endotransglucosylase gene was up-regulated. In Sal vs. CK, the change in methylation level did not cause the difference in the expression level of the gene. The differential methylation site was likely caused by the stress of high pH environment, which in turn regulated the expression and transcription of the gene. Based on our findings and previous studies, we propose a model for the response of *G. barbadense* hypocotyl elongation to sodium stress (Figure 9). The response of plant hormones to sodium enhanced our understanding in the epigenetic mechanism of hypocotyl elongation of *G. barbadense* under sodium stress.

## Discussion

DNA methylation is an important modification of epigenetics, and many of studies have confirmed that it plays an important regulatory role in various stress response processes. WGBS technology can distinguish and determine methylation patterns in single nucleotide units. Genome-wide methylation studies have shown that there is a more subtle relationship between DNA methylation and gene transcription. Many studies have suggested that the dynamic changes of DNA methylation of specific genes are directly involved in the regulation of different stress responses. DNA methylation mediated by RNA-directed DNA methylation (*RdDM*) pathway can dynamically regulate the expression of great amount of heat stress-responsive genes (Popova et al., 2013). In tobacco, changes would occur at the methylation level of glycerophosphodiesterase gene (*NtGPDL*) under aluminum, salt, active oxygen, and other abiotic stresses, especially the demethylation of the GC site of *NtGPDL* in the coding region and promoter. Therefore, the expression of *NtGPDL* was induced, and these results showed the relationship between methylation and *NtGPDL* expression under abiotic stress (Choi and Sano, 2007). Researchers performed low temperature treatment during the dormant period of apple (*Malus domestica*) and found that there was a significant correlation between low temperature environment and methylation changes, and suggested that the stress response mechanism may affect epigenetic regulation through DNA methylation (Kumar et al., 2016). The high expression of GST activity in plants indicated that it contains high levels of ROS (An et al., 2016). Plants eliminated accumulated ROS which caused serious damage to plant cells through antioxidant enzyme systems and secondary metabolites to protect plants from stress. GST-related genes (*GB_A07G2019* and G*B_D05G3291*) were detected in high-level methylation differential regions. Combined with RNA-seq, at the gene expression level, we found differentially expressed genes (*GB_A11G2093*, *GB_A07G2019*, and *GB_D05G3291*) related to GST in the KEGG pathway (Glutathione Movement, Ko00480). GST is synthesized in this pathway and participates in the non-enzymatic pathway of ROS The study provides a dynamic change of epigenetics and transcription in response to high pH sodium salt stress. Previous epigenetic studies on salt stress found that epigenetic regulation genes were up-regulated (Zhou et al., 2007). Studies have confirmed that the methylation level of a species is positively correlated with the genome size of the species (Niederhuth et al., 2016), and it is negatively correlated with the number of genes (Lister et al., 2008; Seymour et al., 2014). Similarly, in our data, the methylation levels at C, CG, CHG, and CHH sites were 19.74, 71.03, 53.87, and 6.26%, respectively. Under the stress of two sodium salts, CG and CHG, the methylation level of the three sequence contexts of CHH and CHH also increased in comparison with the control (Table 1). The occurrence of cytosine methylation was associated with its nearest sequence context. In the three sequence contexts (CG, CHG, and CHH) and the difference between high and low methylation levels, the position of mC was different. According to research reports, this sequence preference had certain differences in different treatments and samples (Lu et al., 2017; Zhang et al., 2020). And our data showed that the methylation level of CHH context changes greater than the other two sequences, and the methylation level in the three sequence contexts was negatively correlated with the number of genes (Figure 2B).

DNA methylation is an important epigenetic trait. During the growth and development of plants, it plays an important regulatory role and is indispensable for maintaining the normal growth and development of plants. Changes in DNA methylation across the whole genome of *Arabidopsis thaliana* during long-term zinc deficiency (Chen et al., 2018). The results of the study proved that the differential DNA methylation in CG and CHG was related to the up-regulation of some zinc deficiency genes, while CHH had no differential DNA methylation. Further studies have found that in the mutant ddc (drm1, drm2, and cmt3) lacking CG methylation, showing more serious developmental defects when zinc deficiency (Chen et al., 2018). At certain stages of plant growth and development, DNA methylation played a crucial role in regulating the expression of key genes or responding to adversity stress (He et al., 2017; Shafiq et al., 2019). It was inferred that plants can regulate gene expression by changing the methylation levels or patterns of certain genes in certain pathways related to stress, which maybe a mechanism to cope with stress. The study of DNA methylation in human tumor cells has found an important way to inhibit gene transcription, which was because abnormal methylation mainly existed in the CpG dinucleotide in the promoter region (Hua et al., 2015). In this study, we conducted a comprehensive analysis of DNA methylation and gene transcription and expression during hypocotyl elongation, and found that the methylation level of the promoter region was higher than that of the gene body (Figure 4D). Our results, demonstrated that DNA methylation in the transcription termination region and the transcription expression regions may inhibit gene expression. This finding is consistent with previous rice methylome research and analysis (Li et al., 2012). In tea plants, CHG and CHH methylations in the promoter positively regulate gene expression, but negatively regulate expression in the gene body. In addition, promoter and downstream non-CG methylation of the tea plant could also positively regulate gene expression. It can be seen that the methylation level of the promoter region played an important role in regulating gene expression, and it was the same in cotton here (Tong et al., 2021). Therefore, DNA methylation in the promoter region is an effective means for gene regulation in response to the environmental adversity.

The TE is widely distributed in plant genomes. Transposons can jump from one position to another on a chromosome, or from one chromosome to another through a series of processes such as cutting and reintegration, thereby inactivating the gene function at the insertion site and forming a mutation (Varagona et al., 1992). Most DNA methylation occurs in heterochromatin regions rich in transposons, but studies of genome-wide methylation profiles have shown that 20% of important gene regions [expressed, non-overlapping genes free of known transposable elements (TE)] were also methylated (Zilberman et al., 2007). Research of anthers found DMRs in the CG and CHG methylation contexts were randomly distributed, but distributed uniformly across the chromosomes in the CHH context. The hypermethylation in the CHH context occurred preferentially in the euchromatin-preferential TEs, which may have caused the unusual CHH methylation pattern detected in anthers (Ma et al., 2018). DNA hypermethylation that occurs in TE can be explained by maintaining chromosome structure and gene stability or protecting the genome from changes by TEs (Saze and Kakutani, 2011). TEs in apples were demethylated under water deficit stress (Xu et al., 2018). Studies in tea plants, corn and other crops found that the level of DNA methylation was negatively correlated with gene density and positively correlated with TE density (Niederhuth et al., 2016; Zhang et al., 2020). The researchers found a significantly low proportion of 24-nt siRNAs in long TEs when comparing their genomic ratio with short Tes, suggested that DNA hypermethylation in long TEs was not predominantly mediated by the RdDM pathway. The H3K9me2-dependent pathway played a prominent role in mediating DNA hypermethylation in long TEs (Wang et al., 2016). In our study, the methylation level of TE may have a certain relationship with the stress treatment. However, how TE responded to the salt-alkaline stress through changes in methylation level remains to be explored further. The methylation levels of TEs in the three sequence contexts were quite different. TEs showed high methylation levels in the context of CG and CHG sequences (Figure 3C), this was consistent with the results of the methylation study of cotton anthers (Zhang et al., 2020).

Living under various adverse conditions, plants can restore and rebuild cell homeostasis through a variety of gene regulation mechanisms. Studies have shown that DNA methylation plays a vital role in plant abiotic stress response, enabling plants to survive in harsh environments. Plant hormones have no obvious specificity. Under adverse stress, plants can adapt to the unfavorable environment by changing their own endogenous hormone levels. They can respond to different regulatory mechanisms to regulate plant growth under various stresses (Zorb et al., 2013; Li et al., 2015). Our results, showed about 100 hormone-related genes whose methylation and expression levels changed (Figures 8B,C). There were relevant genes of IAA, GA3, Brassinolide (BR) and other pathways that responded to the dynamic changes of Na^+^ stress. The methylation level of these genes changed accordingly as well. When the concentration of IAA in plants increased, GH3 protein promoted the combination of free IAA and amino acids, and then degraded through degradation pathways to maintain the dynamic balance of IAA and realized its regulation on the growth of hypocotyl cells (Zheng et al., 2016). Previous studies have found that GA enhances the methyl esterification of pectin and promotes cell elongation by changing the arrangement direction of cell microtubules (Sauret-Güeto et al., 2012). GA mainly affected the elongation of hypocotyl cells by regulating the content of DELLA protein (de Lucas and Prat, 2014). BR and soluble carbohydrates synergistically up-regulated the expression of two TFs, *BZR1* and *BES1* (bri1-EMSSUPPRESSOR 1), thereby promoting hypocotyl elongation (Zhang et al., 2015). The large changes in the expression of methylation-regulated genes shown in our data indicate that the DNA methylation of the genome may be changed due to the response to salt-alkali stress. We believe that methylation and the transcriptional expression of hormone-related genes simultaneously responded to the elongation of the hypocotyl (Figures 1A,C).

The *small auxin upregulated RNA* (SAUR) gene reacted quickly to changes in auxin through dynamic changes, and played an important role in plant dynamic regulation and adaptive growth. The SAUR gene was first discovered in soybean hypocotyls (McClure and Guilfoyle, 1987), and then identified and isolated in Arabidopsis (Hagen and Guilfoyle, 2002), rice (Jain et al., 2006), Populus euphratica (Hu et al., 2018), and other plants. Previous studies have shown that overexpression of some members of the SAUR family can promote cell enlargement and hypocotyl elongation (Chae et al., 2012). These SAURs can indirectly promote the phosphorylation of PP2C-D substrate membrane H^+^-ATPases by inhibiting the protein phosphatase activity of PP2C-D, such as *Arabidopsis* H^+^-ATPase2 (AHA2), the second to last Thr of the self-inhibition domain. Phosphorylation of residues increases the activity of plasma membrane H^+^-ATPases, decreases the extracellular pH, softens the cell wall and makes the cell easy to elongate (Spartz et al., 2014). At high temperatures, SAUR gene expression can be relied on to promote hypocotyl elongation (Franklin et al., 2011). Under the stress of Na^+^, in our study, the expression of *G. barbadense* hypocotyls *GbSAUR39, GbSAUR50, GbSAUR66, GbSAUR67, GbSAUR72* and other genes significantly decreased, which also explains the inhibition of cell elongation of the hypocotyl under stress (Figures 1B,C). We found that the TF *TIFY* of the *GbCXE* gene, was closely related to abiotic stress. The gene was significantly up-regulated, indicating that there is a complex regulatory mechanism related to hormones and TFs after the stress to cotton hypocotyl.

As reported in the literature, the inactive indole acetic acid (IAA) in plants is stored in the form of sugar ester methyl esters, etc., and carboxylesterase (CXE) can act on these sugar ester methyl esters to generate active auxins, thereby maintaining the balance of plant growth and development (Kowalczyk et al., 2003; Woodward and Bartel, 2005). CXE was found in immature endosperm tissue of maize to regulate the metabolism of IAA, and CXE in maize can also regulate the metabolism of gibberellin (GA20) glycosyl (Schneider et al., 1992). Auxin could affect cell wall ductility and turgor pressure, and enhanced cell wall plasticity (Takahashi et al., 2012). This report shows that plant CXE played an important role in the regulation of plant hormone activity, so that plants could respond to changes in the external environment. In our stress treatment the *GBXTH22* gene was significantly up-regulated under high pH Na^+^ treatment, this observation might suggest that the gene was in a state of hypomethylation. When the tomato *LeXTH1* gene was studied by transgenic technology, it was found that the tomato *LeXTH1* gene was specifically expressed in the upper end of the tomato seed hypocotyl, and a soluble protein transcribed and translated by it was related to the relaxation and extension of the hypocotyl cell wall structure, which proved its important role in tomato seed germination (Miedes et al., 2011). XTH is an important cell wall modification enzyme that catalyzes the cleavage and regeneration of xyloglucan. Existing research suggests that xyloglucan endoglycosylase (XET) was very important for dispersing cell walls, which plays a major role in cell elongation (Cosgrove, 2005).

During the elongation and growth of lettuce and cucumber hypocotyls and pea internodes induced by gibberellin GA3, the enzyme activity of XET was significantly enhanced (Potter and Fry, 1993; Ian and Fry, 2010). Experimental results shown that XTH involves in cell expansion and elongation caused by cell wall relaxation during plant growth. In *Arabidopsis*, BR promoted cell wall extension by increasing *XET* expression, and *AtXTH22* gene (*TCH4*) promoted cell expansion and extension in *Arabidopsis* (He et al., 2003). Based on our results and previous reports, we proposed a model of hypocotyl elongation mechanism under Na^+^ stress (Figure 9). The mechanism of cell elongation is mediated by phytohormonal pathways represented by IAA, GA, and BR. In this model, although the mechanism how SAUR protein, XTH22 protein and hormones act is still not completely clear, we have determined that XTH22 has some genes at a low methylation level. The molecular mechanism of how hormones and DNA methylation regulate the expression of *XTH* genes is worthy of further investigation.

## Conclusion

*Gossypium barbadense* is a cultivated cotton. It is not only known for producing high-quality fiber, but also has salt and alkali resistance. It is a pioneer crop in saline and alkaline soils. However, the growth and yield of cotton will be severely restricted by external and internal environmental factors. In this study, a cytosine methylome map of cotton hypocotyl was developed for the first time to learn the cotton response to methylation for regulating cell elongation under the stress. Detailed analysis of DNA methylation and gene transcription reveals a regulatory mechanism of gene promoter methylation in response to the stress during cotton hypocotyl elongation. Such methylation is found to regulate cell extension actively at seed germination stage. Methylation regulation under the stress is likely involved in plant hormone signal transduction. The study provides new insights for plant biologists to further examine the regulatory mechanism of plant abiotic stress response with the methylation. Ultimately the information and resources obtained from the study will facilitate cotton breeders in developing tolerant cotton cultivars that can be grown under adverse environments.

## Data Availability Statement

The datasets presented in this study can be found in online repositories. The names of the repository/repositories and accession number(s) can be found below: NCBI SRA Bio Project, PRJNA734700 and PRJNA735131.

## Author Contributions

CR, WY, WG, and JY designed the study, interpreted the results, and wrote the manuscript. CR prepared the materials and conducted the experiments. CR, YZ, and YF collected and analyzed the experimental data. MH, MD, QW, XC, XL, DW, and SW provided technical assistance and research support. All authors contributed to the article and approved the submitted version.

## Conflict of Interest

The authors declare that the research was conducted in the absence of any commercial or financial relationships that could be construed as a potential conflict of interest.

## Publisher’s Note

All claims expressed in this article are solely those of the authors and do not necessarily represent those of their affiliated organizations, or those of the publisher, the editors and the reviewers. Any product that may be evaluated in this article, or claim that may be made by its manufacturer, is not guaranteed or endorsed by the publisher.

## Footnotes

1
http://www.bioconductor.org/packages/release/bioc/html/ballgown.html

2
http://www.bioinformatics.babraham.ac.uk/projects/fastqc/

## Abbreviations

WGBS: Whole Genome Bisulfite Sequencing
BS: Bisulfite Sequence
TE: transposable element
TF: transcription factor
*G. barbadense*: *Gossypium barbadense*
CRY: *cryptochrome*
LHY: *LATE ELONGATED HYPOCOTYL*
CO: *CONSTANS*
NEC: non-embryogenic callus
SE: somatic embryos
SRA: successful regeneration acclimation
FPKM: fragments per kilobase of exon model per million mapped fragments
GO: Gene ontology
KEGG: kyoto encyclopedia of genes and genomes
DEG: differentially expressed gene
DMG: differentially methylated gene
DMR: differentially methylated regions
TSS: transcription start site
TES: transcription end site
GA: gibberellin
ABA: abscisic acid
JA: jasmonic
SA: salicylic acid
BR: brassinolide
*SAUR*: *small auxin upregulated RNA*
XTH: *Xyloglucan Endotransglucosylase/hydrolase*
CXE: *carboxylesterase*.
