# Supplementary material for: Insight Between the Epigenetics and Transcription Responding of Cotton Hypocotyl Cellular Elongation Under Salt-Alkaline Stress
Source: Front Plant Sci. 2021 Nov 11;12:772123. doi: 10.3389/fpls.2021.772123 (PMC8632653; doi:10.3389/fpls.2021.772123)
Supplement: Supplementary file 1 [file Data_Sheet_1.docx]

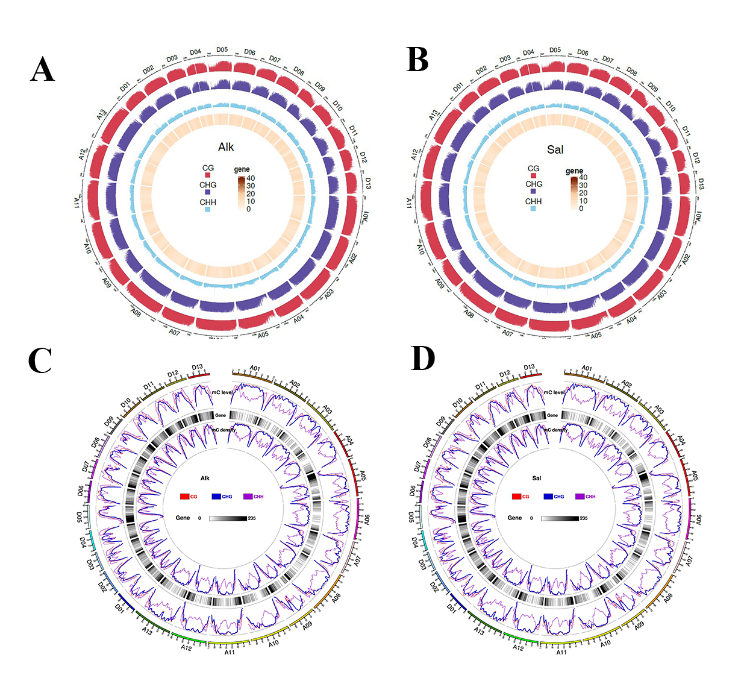


Fig.S1 Epigenome of *G. barbadense.*

A, B. The distribution circle map of methylation sequencing data within the chromosome range (Alk. Sal). (1) The outermost circle is a scale based on the length of the corresponding chromosome; (2) The following three circles (from outside to inside) are respectively the methylation background display of CG, CHG, CHH in the corresponding chromosome interval of the PF group (corresponding to red, purple and blue respectively, the denser the color line indicates the higher the methylation background level); (3) The innermost circle indicates the corresponding the number of genes in the interval, the darker the color indicates the greater the number of genes in the area.

C, D. Circos plot showing the level and density of 5-mCs in the three sequence contexts and the gene density for all 26 chromosomes of the cotton genome. For the line graph of the level and density of 5-mCs, the CG, CHG, and CHH sequence types are displayed as red, blue, and purple lines, respectively. (Alk. Sal)


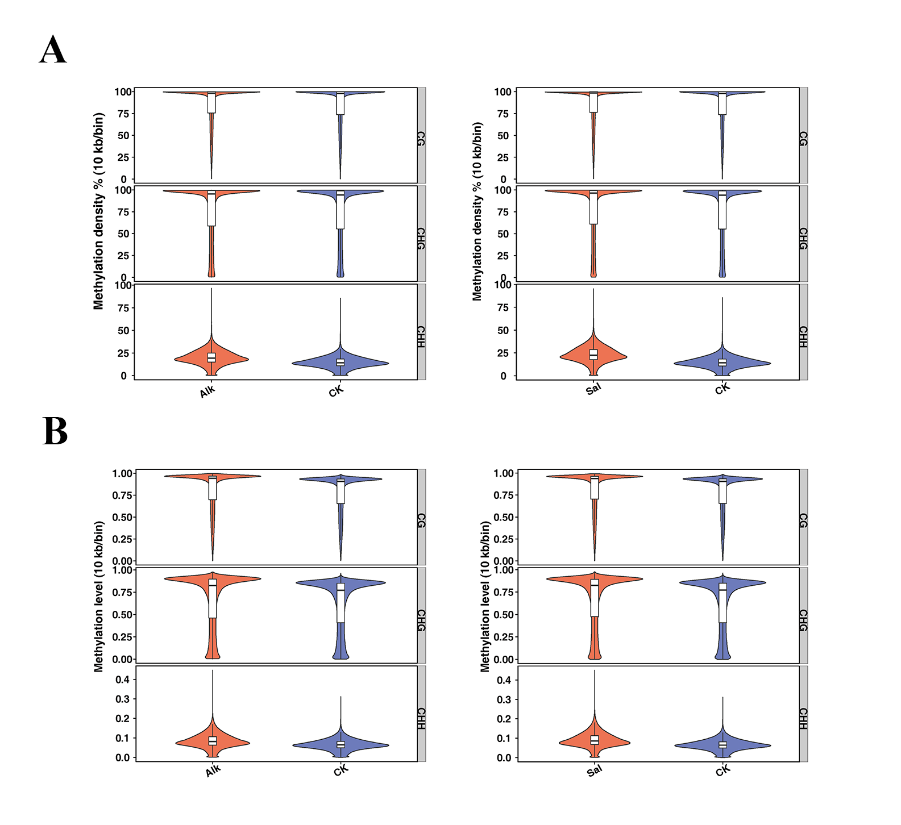


Fig.S2 Violin maps of whole genome-wide methylation levels (MLs) and densities for three cotton samples.

A. Violin map of whole genome-wide methylation densities in C, CG, CHG and CHH contexts for each of the samples. The X-axis represents different sample names; the Y-axis represents the percentage of mC densities among the corresponding cytosine sites (10 Kb/bin).

B Violin map of whole genome-wide MLs in C, CG, CHG and CHH contexts for each of the samples. The X-axis represents different sample names; the Y-axis signifies MLs (10 Kb/bin) in different sequence contexts and the width of each violin represents mC abundance at the corresponding ML.The width of each violin represents the mC abundance at the corresponding methylation density.


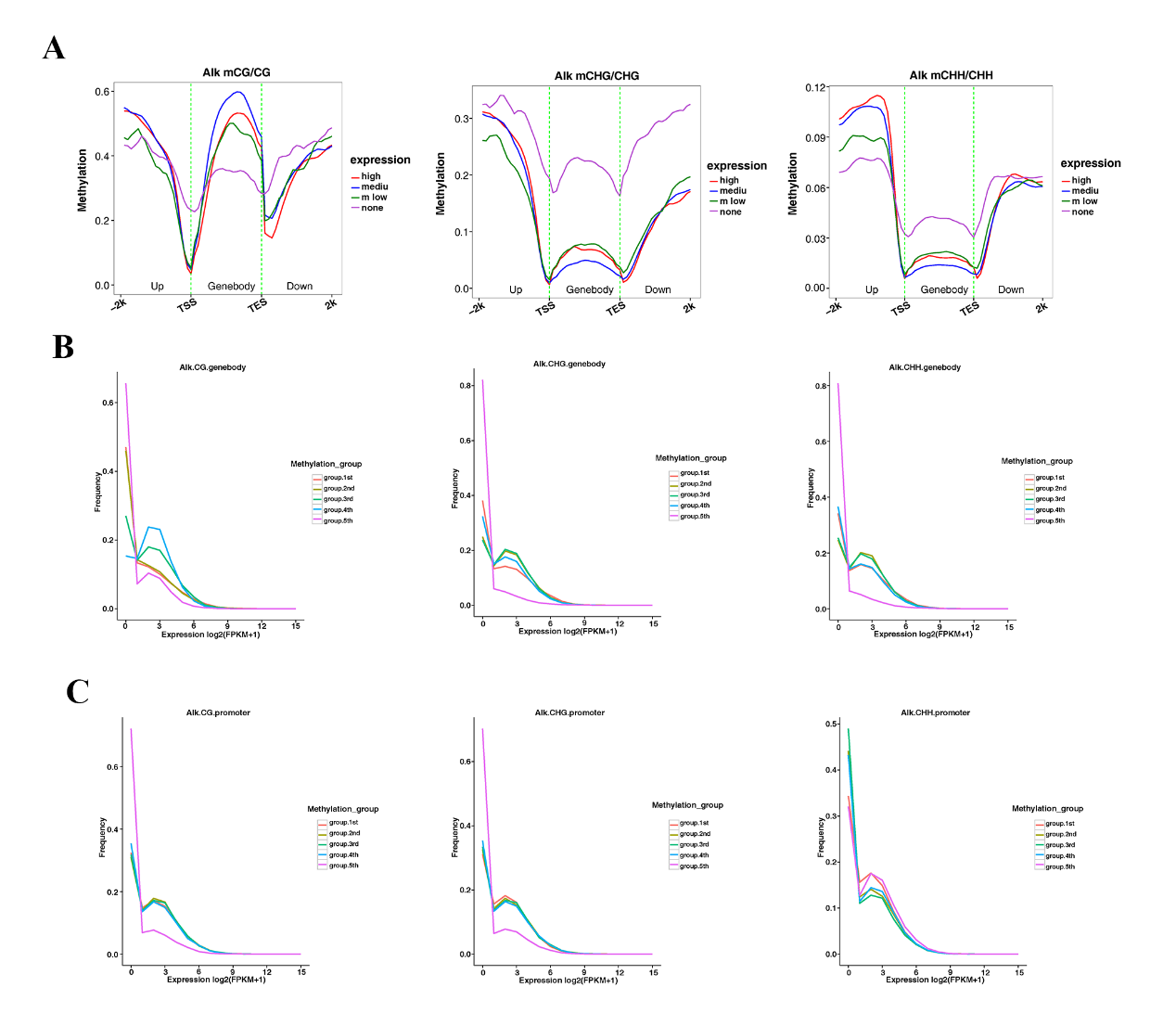


Fig.S3 Relationship between methylation and transcriptome of different expression distribution under alkaline stress.

A. The distribution of methylation levels of genes under different expression levels in the genebody and its upstream and downstream 2Kb regions under different sequence contexts. (The abscissa represents different regions, and the ordinate represents the methylation level. Different colors represent different expression levels)

B, C, The expression levels of genes under different methylation levels in the genebody and promoter regions under different sequence contexts. (The abscissa represents the expression level, and the ordinate represents the frequency of the gene at the corresponding expression level. Different colors represent different methylation grade).


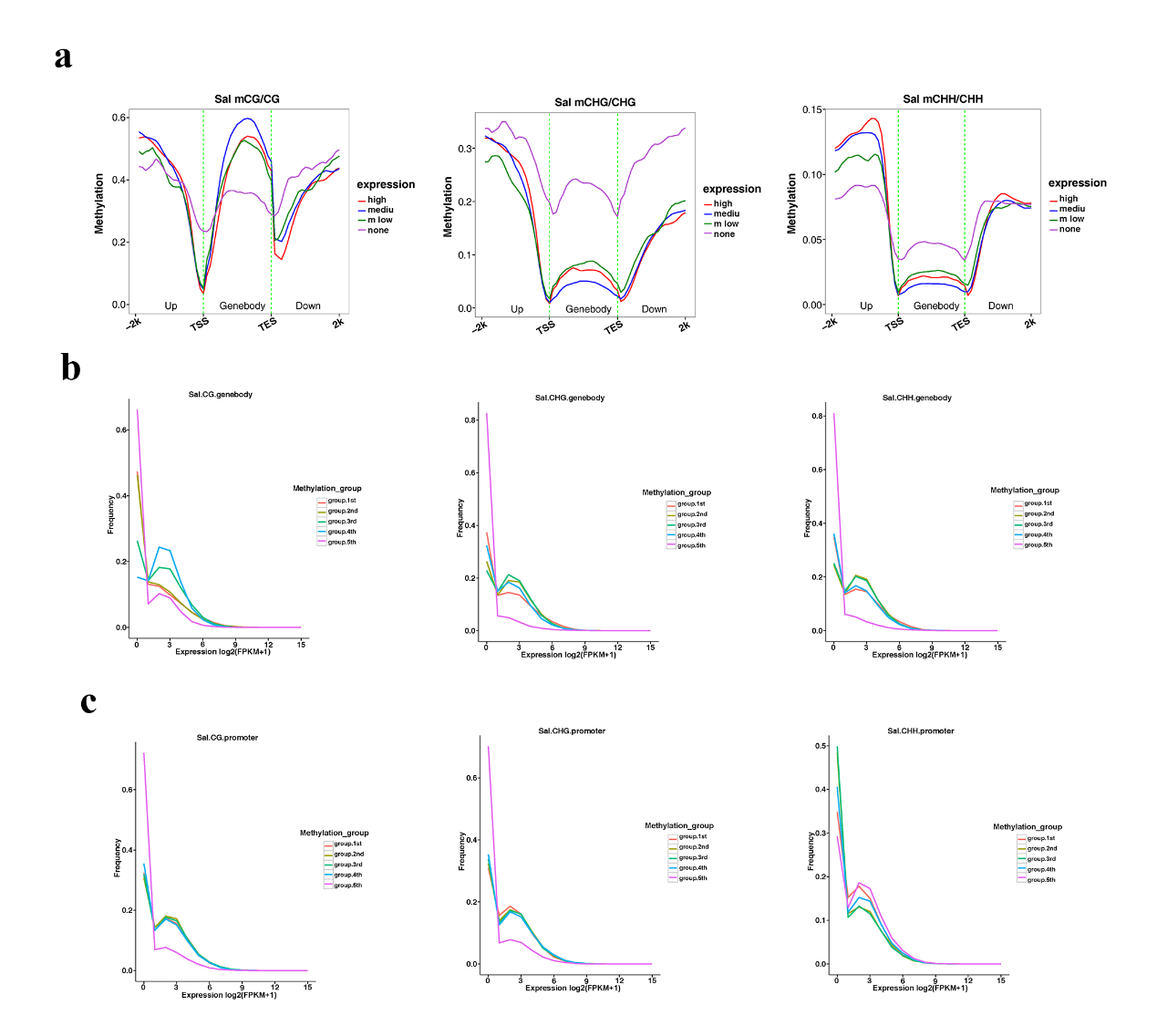


Fig.S4 Relationship between methylation and transcriptome of different expression distribution under salt stress.

A. The distribution of methylation levels of genes under different expression levels in the genebody and its upstream and downstream 2Kb regions under different sequence contexts. (The abscissa represents different regions, and the ordinate represents the methylation level. Different colors represent different expression levels)

B, C, The expression levels of genes under different methylation levels in the genebody and promoter regions under different sequence contexts. (The abscissa represents the expression level, and the ordinate represents the frequency of the gene at the corresponding expression level. Different colors represent different methylation grade).


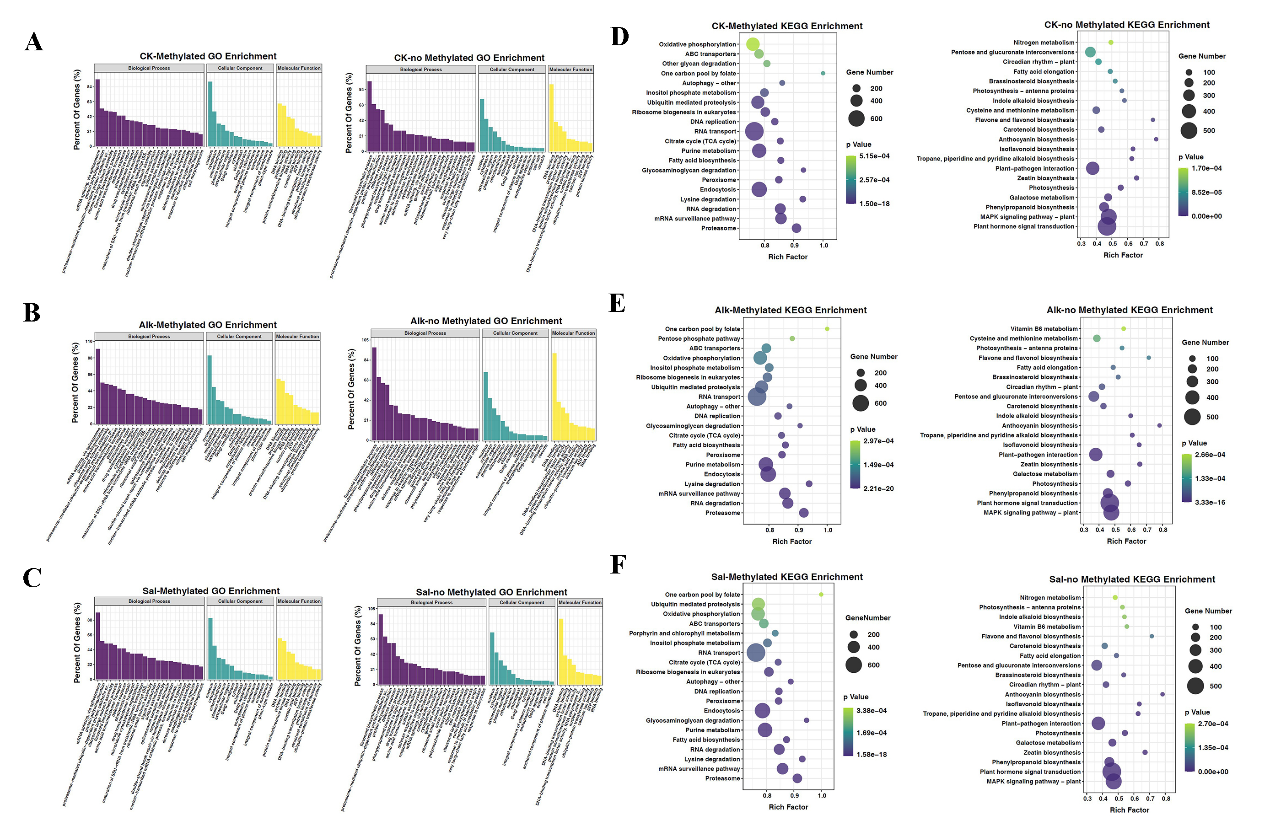


Fig.S5 Enrichment analysis of methylated and unmethylated genes.

A, B, C. Gene Ontology (GO) enrichment analysis of all DMGs.

D, E, F. KEGG pathway enrichment of hypermethylated and hypomethylated genes in GB under salt and alkaline stress. The size of the circle represents gene numbers, and the colour represents the p-value.


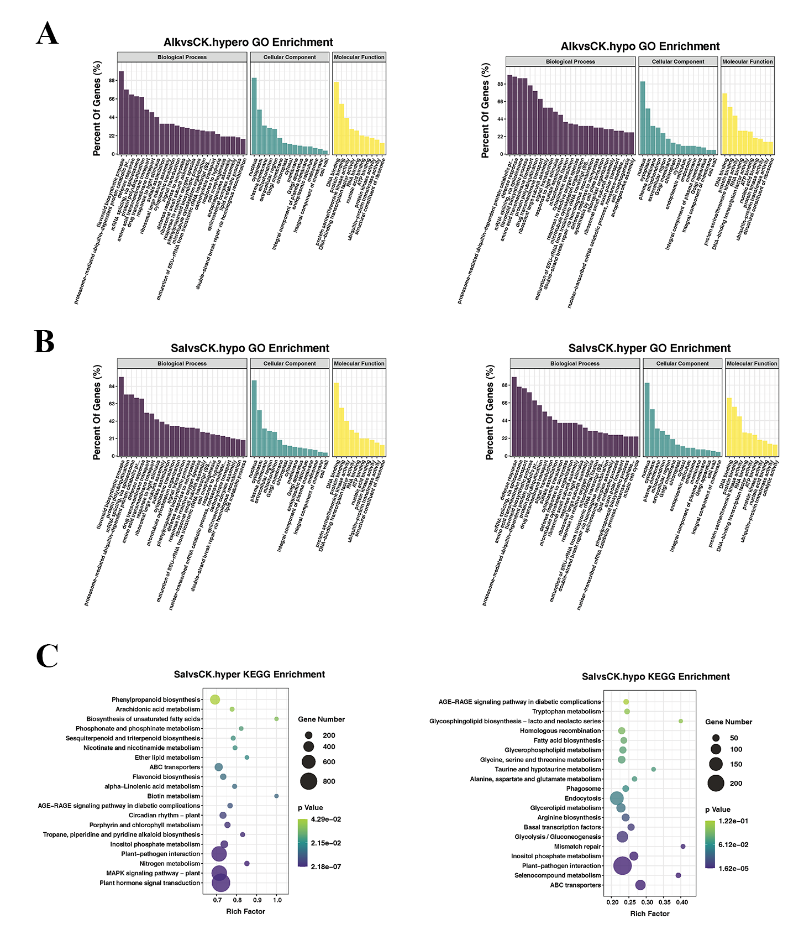


Fig.S6 Enrichment of hypermethylated and hypomethylated genes.

A, B, Gene Ontology (GO) enrichment analysis of all DMGs.

C, KEGG pathway enrichment of hypermethylated and hypomethylated genes in GB under salt and alkaline stress. The size of the circle represents gene numbers, and the colour represents the p-value. AlkvsCK: alkaline stress versus control; SalvsCK: salt stress versus control.


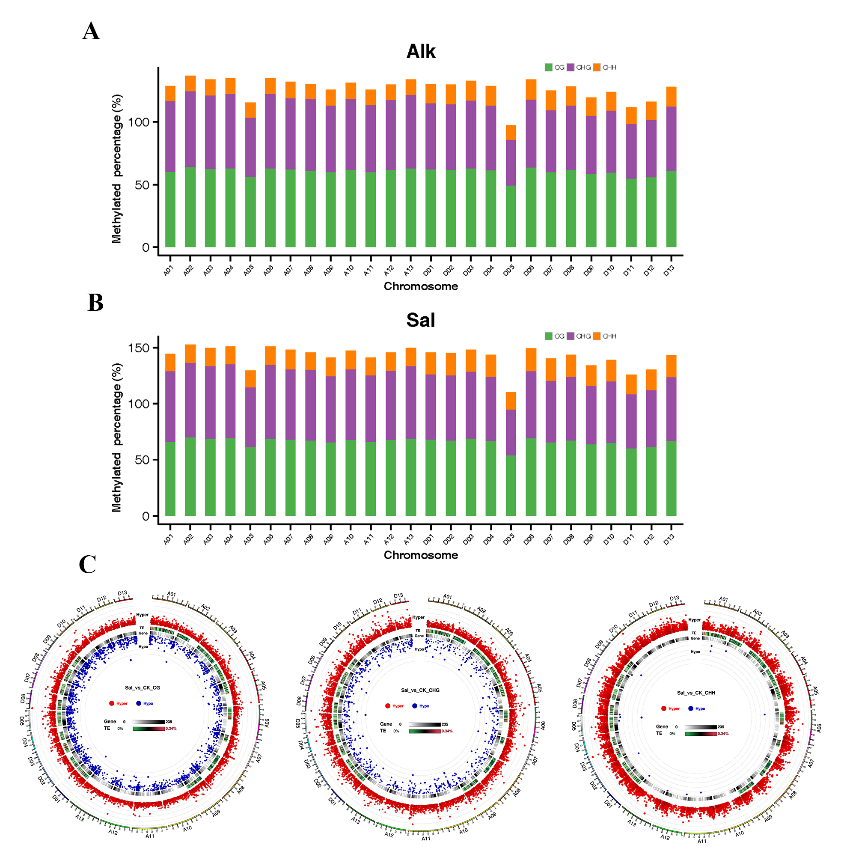


Fig.S7. Dynamic changes of DNA methylation under alkaline stress.

A, B, The ratio of methylated C sites to the total C sites on the chromosome in different sequence contexts (Alk, Sal). Different colors represent methylated C sites under different contexts, and the length of each column represents the percentage of the sequence context methylation sites in the sequence context of the chromosome.

C, The overall Circos diagram of DMR in 3 sequence contexts (CG/CHG/CHH). Graphic display instructions, from the outside to the inside: (1) Hyper DMR statistical value log5 (|areaStat|); the higher the outer dot, the larger the position difference is, and it is indicated by the red circle, (2) TE original proportion heat map (the color indicates the scale), (3) Gene density heat map (the color indicates the scale), (4) Hypo DMR statistical value log5 (|areaStat|); the higher the inward dot, the more significant the position difference, the blue circle indicates.


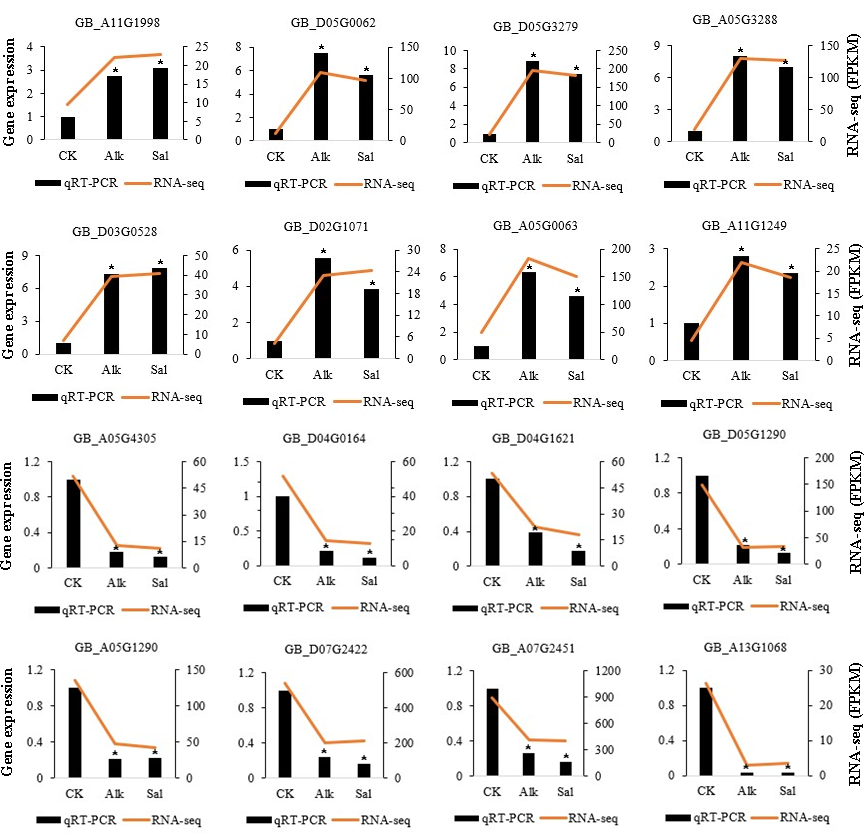


Fig.S8. The qRT-PCR for differentially expressed genes (The left y-axis represented qRT-PCR data, and the right y-axis represented FPKM in RNA-seq data, *P <0.05).
